# Systematic Aβ Analysis in *Drosophila* Reveals High Toxicity for the 1-42, 3-42 and 11-42 Peptides, and Emphasizes N- and C-Terminal Residues

**DOI:** 10.1371/journal.pone.0133272

**Published:** 2015-07-24

**Authors:** Maria Jonson, Malgorzata Pokrzywa, Annika Starkenberg, Per Hammarstrom, Stefan Thor

**Affiliations:** 1 Department of Clinical and Experimental Medicine, Linkoping University, SE-581 85, Linkoping, Sweden; 2 Department of Physics, Chemistry and Biology, Linkoping University, SE-581 83, Linkoping, Sweden; National Center for Geriatrics and Gerontology, JAPAN

## Abstract

Brain amyloid plaques are a hallmark of Alzheimer's disease (AD), and primarily consist of aggregated Aβ peptides. While Aβ 1-40 and Aβ 1-42 are the most abundant, a number of other Aβ peptides have also been identified. Studies have indicated differential toxicity for these various Aβ peptides, but *in vivo* toxicity has not been systematically tested. To address this issue, we generated improved transgenic *Drosophila UAS* strains expressing 11 pertinent Aβ peptides. *UAS* transgenic flies were generated by identical chromosomal insertion, hence removing any transgenic position effects, and crossed to a novel and robust *Gal4* driver line. Using this improved *Gal4/UAS* set-up, survival and activity assays revealed that Aβ 1-42 severely shortens lifespan and reduces activity. N-terminal truncated peptides were quite toxic, with 3-42 similar to 1-42, while 11-42 showed a pronounced but less severe phenotype. N-terminal mutations in 3-42 (E3A) or 11-42 (E11A) resulted in reduced toxicity for 11-42, and reduced aggregation for both variants. Strikingly, C-terminal truncation of Aβ (1-41, -40, -39, -38, -37) were non-toxic. In contrast, C-terminal extension to 1-43 resulted in reduced lifespan and activity, but not to the same extent as 1-42. Mutating residue 42 in 1-42 (A42D, A42R and A42W) greatly reduced Aβ accumulation and toxicity. Histological and biochemical analysis revealed strong correlation between *in vivo* toxicity and brain Aβ aggregate load, as well as amount of insoluble Aβ. This systematic *Drosophila in vivo* and *in vitro* analysis reveals crucial N- and C-terminal specificity for Aβ neurotoxicity and aggregation, and underscores the importance of residues 1-10 and E11, as well as a pivotal role of A42.

## Introduction

Alzheimer’s disease (AD) is the most common neurodegenerative disorder, and the fact that AD is an age-dependent disease with high age as the primary risk factor makes the global increased lifespan an exacerbating factor [1]. AD is characterized by the formation of extracellular Amyloid β (Aβ) plaques [2, 3] and intracellular Tau tangles in the brain of patients [4]. The amyloid plaques consist of different variants of the Aβ peptide, with the most abundant being Aβ 1–40 and Aβ 1–42. An overwhelming number of studies, using a multitude of approaches, has resulted in the general view that 1–40 is slowly aggregatory and has low toxicity in vivo, while 1–42 is very aggregation prone and highly toxic [5]. However, due to sequential cleavage of the Amyloid-β Precursor Protein (APP), by β- and γ-secretase, and proteolytic processing, a complex pool of additional Aβ peptides (31–46 amino acids long) is generated. Several studies provide increasing evidence that such variable Aβ peptides may be significant in AD pathogenesis [6–9]. However, the *in vivo* toxicity of these Aβ peptides has not been addressed in a rigorous and comparative manner.

Over the past decade *Drosophila melanogaster* (*Drosophila*) has emerged as a model system for various neurodegenerative disorders, among them AD [10, 11]. Expression of the Aβ peptide in the *Drosophila* model system results in shortened lifespan, protein accumulation, impaired locomotor behavior and amyloid build-up [12–14]. These are all hallmarks of AD, and because many of these hallmarks are not always readily detected in rodent models of the disease [15] *Drosophila* may offer some advantages as a supplementary model. In addition, novel landing site technology [16, 17] developed in *Drosophila* allows for a set of transgenic constructs to be inserted at one specific and pre-determined location. This transgenic system, currently unique to *Drosophila*, now allows for systematic *in vivo* toxicity testing of Aβ peptides. Previous *Drosophila* models of Aβ 1–42 toxicity showed rather mild effects on lifespan when compared to controls [10, 11, 18]. This makes both systematic structure-function analyses of Aβ and compound testing slow and difficult to evaluate. To facilitate studies of Aβ in the fly model we aimed to increase Aβ expression levels, thereby hoping to reduce lifespan in this model. This was done by generating a stronger and more persistent *Gal4* line, as well as generating stronger *UAS* transgenes, paying attention to three important aspects: signal sequence, codon optimization and start codon sequence.

Using the improvements of landing site technology and optimized *Gal4/UAS* transgenes, we have addressed Aβ peptide neurotoxicity. With this system we observe greatly reduced lifespan and locomotor activity in the Aβ 1–42 transgene, when compared to previous systems. We generated 10 additional transgenic *Drosophila UAS* strains, expressing pertinent N-terminal and C-terminal variant peptides in the central nervous system of the fly, as well as five specific amino acid (aa) mutants. We find that the N-terminal truncations 3–42 and 11–42 are highly toxic, and that mutating the N-terminal residues (E3A and E11A) provides support for toxicity of E11, and partly for E3. In addition, we find compelling evidence that C-terminal A42 is pivotal for high toxicity, whereas extensions to 1–43 show lower toxicity. Toxicity was generally correlated with extensive Aβ aggregation in the brain and insolubility in immunoassay. Our results support the view that several Aβ variants, in addition to 1–42, are neurotoxic, and that this improved *Drosophila* model may be useful for addressing proteo-toxicity.

## Results

### Generation of a stronger and postmitotic *Gal4* line, with more persistent adult expression

The majority of previous studies addressing toxicity of human neurodegenerative disease proteins in *Drosophila* have used the *C155-elav-Gal4* driver line, which expresses *Gal4* broadly in the nervous system [19]. However, we noticed four apparent shortcomings of this driver line: 1) It also expresses *Gal4* in progenitor cells (also noted by others [20]); 2) its expression declines in adult flies; 3) compared to some more restricted *Gal4* lines, such as *apterous-Gal4*, *C155-elav-Gal4* is not strongly expressed in each individual neuron (not shown); 4) due to its insertion on the X chromosome, males (which in *Drosophila* shows X chromosome activation) express considerably higher levels (S1A–S1F Fig). To address these four problems we turned to a different driver, *n-syb-Gal4*, where Gal4 expression is under the regulatory control of the *neuronal-synaptobrevin* (*n-syb*) gene (kindly provided by J. Simpson). By analyzing expression of *UAS-eGFP*, we found that in contrast to *C155-elav-Gal4*, which is active already at embryonic stage 11 i.e., during neurogenesis, the *n-syb-Gal4* driver did not turn on until at embryonic stage 17 i.e., after neurogenesis (not shown). In addition, analysis of adult flies revealed no sign of down-regulation of GFP in three weeks old fly brains (S1G–S1L Fig). However, in spite of these advantages, the expression levels of the original *n-syb-Gal4* lines available were not apparently more robust than that of *C155-elav-Gal4*. We thus obtained the *n-syb-Gal4 P* element construct (also kindly provided by J. Simpson) and generated 62 new, randomly inserted, transgenic lines. Crossing these to *UAS-eGFP* allowed for rapid analysis of expression levels in adult brains. Strong inserts on the 2^nd^ or 3^rd^ chromosomes were combined by recombination, and two strong multi-insert driver lines were developed; one for the 2^nd^ and one for the 3^rd^ chromosome. For the majority of experiments in this study, a composite driver on the 3^rd^ chromosome was used, hitherto simply referred to as *n-syb-Gal4*. Comparing the new *n-syb-Gal4* to *C155-elav-Gal4* we observed broad eGFP expression in most if not all brain areas containing neuronal cell bodies (S1G–S1L Fig). Expression was stronger and broader than *C155-elav-Gal4*, and in contrast to *C155-elav-Gal4*, expression was not reduced in 10 or 20 day old flies (S1G–S1L Fig). These eGFP expression results were confirmed in a more quantitative assay i.e., western blot (S1M and S1N Fig). We also compared our multi-insert *n-syb-Gal4* driver to the *n-syb-Gal4* driver currently available from the Janelia *Gal4* collection at the Bloomington Drosophila Stock Center. To this end we used both drivers to express Aβ 1–42, and noticed a significantly shorter life-span with our novel driver when compared to the Janelia driver (S1O Fig). We conclude that the new *n-syb-Gal4* multi-insert driver successfully addresses the four shortcomings of the *C155-elav-Gal4* driver, being turned on after neurogenesis, at high levels, expressed at comparable levels in both males and females, and being persistently expressed in adult flies.

### Generation of stronger *UAS-Aβ* transgenic lines

**Fig 1:**
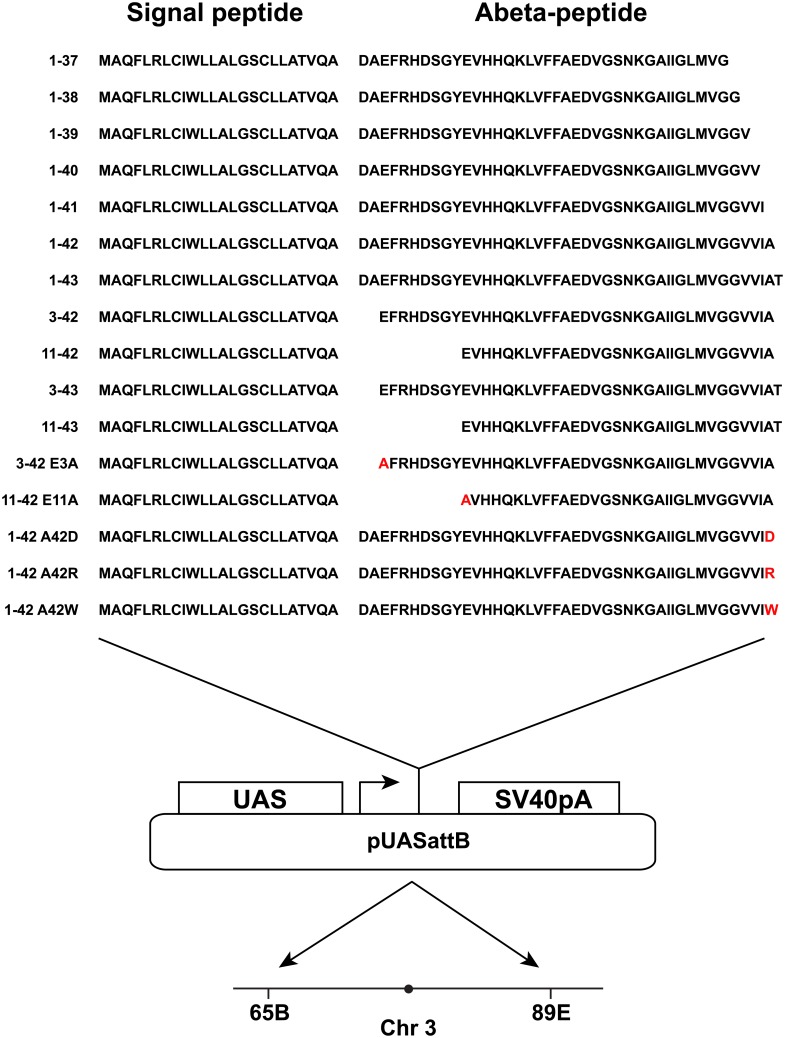
Transgenic set-up for Aβ transgenes. Transgenic design showing the signal- and Aβ-peptide for each peptide studied. DNAs were cloned into the pUASattB vector and integrated at landing-site position 65B and 89E, using phiC31 transgenesis.

Previous studies expressing various Aβ peptides in *Drosophila* did not involve fully optimizing the transgenic constructs for high-level expression [18, 21]. To this end we designed new Aβ constructs, paying attention to three important aspects: signal sequence, codon usage and start codon sequence (see Materials and Methods). The novel Aβ constructs, denoted *UAS-Aβ-X-X*, were inserted into the pUASattB vector [16] and landed at position 65B or 89E on chromosome 3 (Fig 1 and S1 Suppl. Info.).

**Fig 2:**
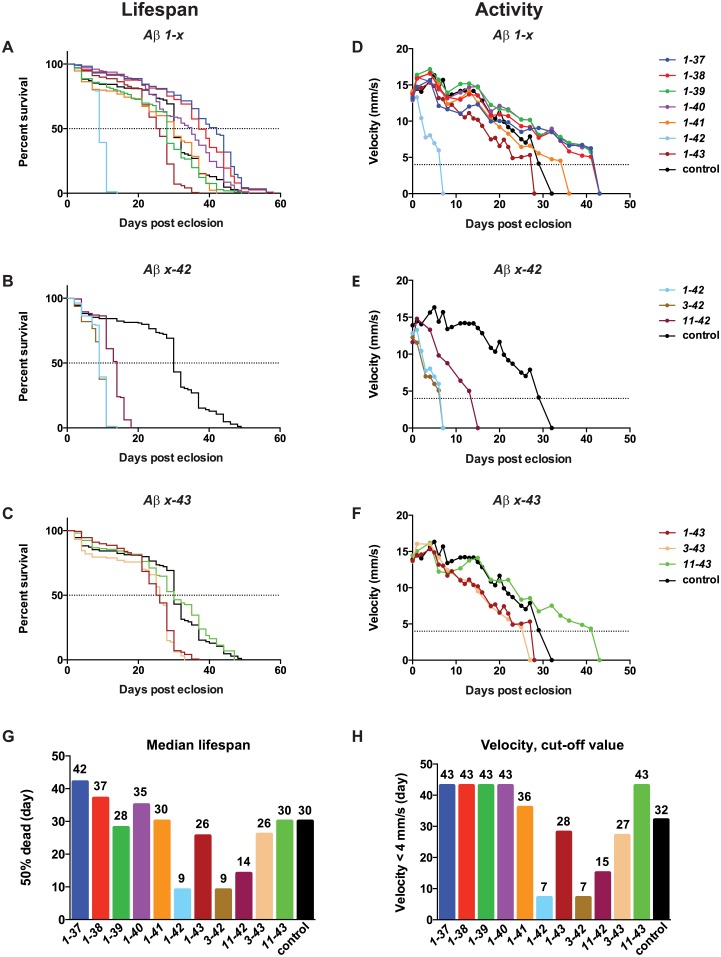
Expression of Aβ peptides ending at amino acid 42 shortens lifespan and impairs locomotor activity. (A-C) Lifespan trajectories of *Drosophila* flies expressing Aβ in the nervous system. (D-F) Locomotor activity analyzed by velocity using iFly. (G) Summary of the median lifespan (50% dead). (H) Summary of the day the velocity reaches the cut-off value of 4 mm/s. Survival plots were calculated using the Kaplan-Meier method. See S1 Table for statistical analyses of differences between the control and all variants.

To test the toxicity of the novel *Gal/UAS* stocks, we first focused on Aβ 1–42. We found that the *n-syb-Gal4/UAS-Aβ-1-42* flies displayed a greatly reduced median lifespan (where 50% of the flies have died), with an average of only 9 days, when compared to the *n-syb-Gal4/+* controls (avg. 30 days) (Fig 2A and 2G). Thus, our new transgenic model expressing wt Aβ 1–42 showed a severely reduced lifespan when compared to our previously published studies [13, 22]. Another added benefit was that, in contrast to *elav-Gal4*, lifespans were not significantly different in males when compared to females (not shown).

### Expression of an Aβ peptide ending at amino acid 42 severely shortens lifespan

Having established a robust Aβ transgenic model with strong phenotype, we systematically investigated the effect on lifespan of a set of pertinent Aβ peptides (Fig 1), all of which have been shown to be amyloidogenic *in vitro*[23]. Survival of the transgenic flies was compared to *n-syb-Gal4/*+ control flies. Focusing first on N-terminal variants, we found that regardless of if the N-terminus started at aa 1, 3 or 11, if the peptide ended at aa 42 it resulted in a strongly reduced lifespan compared to the control flies (Fig 2B and 2G). A longer lifespan was observed for the 11–42 peptide (median lifespan 14 days) compared to the 1- and 3–42 peptides (median lifespan 9 days) (Fig 2B and 2G). A similar pattern was seen in flies expressing an N-terminal truncated peptide ending at aa 43, however, the 11–43 peptide was as healthy as the control, and the 1–43 and 3–43 peptides showed a modest reduction of lifespan compared to the control, with a median lifespan of 25–26 days (Fig 2C and 2G and S1 Table).

We next addressed the lifespan of a set of C-terminal variants, and unexpectedly found that flies expressing any Aβ peptide shorter than 42 aa (1–41, -40, -39, -38, -37) were similar to control flies, having a median lifespan of 28–42 days (Fig 2A and 2G and S1 Table). This is in stark contrast to flies expressing 1–42, which display a median lifespan of 9 days (Fig 2A and 2G). When expressing the 1–43 C-terminal variant, we observe a reduced lifespan compared to the control, however, not to the same extent as for the 1–42 flies (Fig 2A and 2G and S1 Table).

### Expression of an Aβ peptide ending at amino acid 42 results in extensive locomotor dysfunction

The lifespan assay is a straightforward read-out of general organismal protein toxicity. However, to address effects of toxicity upon the function of the nervous system, we turned to the recently developed fly activity imaging system iFly [24]. This system allows for probing the negative geotaxic movement of flies, and to calculate several behavioral parameters using a 3D-tracking algorithm; here, we choose to analyze velocity and angle-of-movement. Velocity describes how fast the flies move from the bottom of the vial to the top of the vial, and as the flies are neurologically impaired the velocity decreases. Shortly before the flies die they become immobile and their velocity cannot be recorded, thus a cut-off value of 4 mm/s was set as an indication of disability. The angle-of-movement describes the deviation from a straight path when flies move from the bottom of the vial to the top. As flies age or get sicker they tend to move in a more disordered pattern, resulting in an increase of the angle-of-movement. This increase indicates that mobility functions, such as orientation and movement direction, of the flies are impaired, and the cut-off value was set to 80°.

Focusing first on N-terminal variants, in agreement with what was observed in the lifespan assay, when the peptide ended at aa 42, N-terminal truncations had minor impact on the toxicity leading to decreased movement. A minor increase in activity was observed in flies expressing the 11–42 peptide compared to the 1–42 and 3–42 flies, but these values are still significantly reduced compared to the control (Fig 2E and 2H). Velocities for flies expressing 1–43 or 3–43 were similar during the course of the experiment, showing a slight decrease compared to the control (Fig 2F and 2H), while flies expressing 11–43 had even higher activity than the control flies. The results from the angle-of-movement analysis were in agreement with the velocity results (S2B Fig).

For C-terminal variants, we found that there was a good correlation between the phenotype seen in the lifespan assay and velocity, as well as angle-of-movement. The apparent discrepancy of total days stems from selection bias of flies to be assayed in the activity assay (see Materials and Methods). Flies expressing any Aβ C-terminal peptide shorter than 42 aa displayed velocity and angle-of-movement similar to control flies, reaching the cut-off value at day 32 or later (Fig 2D and 2H and S2A Fig). Flies expressing a peptide ending at aa 42 showed a similar initial value as the control flies, but a rapid decrease in velocity was observed; already at day 7 the cut-off value was reached and the movement of the flies was so limited that their velocity could not be recorded (Fig 2D and 2H). The same rapid deviation was seen for the angle-of-movement (S2A Fig). 1–43 displayed a decreased velocity compared to the control, reaching the cut-off value at day 28 (Fig 2D and S2C Fig). In summary, the activity results were in general agreement with the lifespan results, demonstrating strong effects of 1–42, 3–42 and 11–42.

### Protein expression and solubility are in agreement with toxicity

Accumulation of aggregates and hence presumed toxicity of Aβ is primarily driven by the protein concentration. Hence, we next addressed Aβ peptide expression concentrations by using a sandwich immunoassay (Meso Scale Discovery; MSD) to detect Aβ peptides in head extracts from 1, 10 and 20 day old adult flies. 1–42, 3–42 and 11–42 expressing flies were only assayed at day 1 and 10, due to their short lifespan.

We used two different antibody set-ups to enable determination of the level of Aβ in the flies. For all flies, detection was achieved by using an antibody recognizing the middle of the peptide (4G8), while as the capture antibody we had to use two variants: peptide ending at aa 42 were captured with an anti-Aβ 42 antibody (12F4), while all other variants, except the 11–43 expressing flies, were captured by a N-terminal antibody (6E10). For accurate concentration determination, each experiment was aligned with a standard curve using synthetic Aβ 1–42 (Meso Scale Discovery, MD, USA) on each 96 well assay plate. These set-ups allowed us to analyze all peptides but the 11–43 expressing flies, since neither the 6E10 nor the 12F4 antibody had binding epitopes within that sequence. We divided our head extracts into a soluble fraction and an insoluble fraction. The soluble fraction was composed of fly heads homogenized in a Hepes buffer (pH 7.3), while the insoluble fraction was obtained from heads homogenized in a Hepes buffer containing 5M GuHCl.

**Fig 3:**
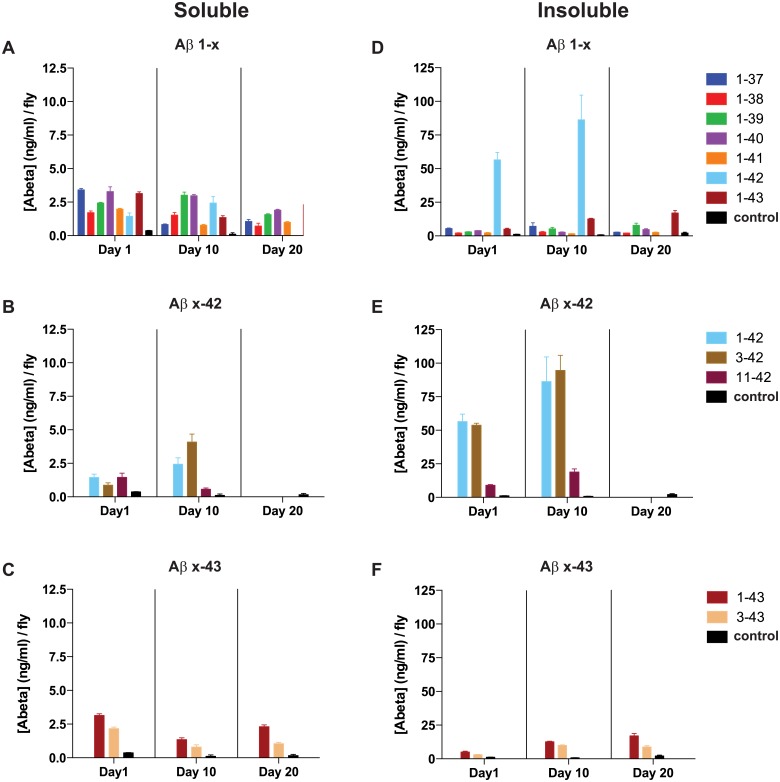
Aβ accumulation and solubility differ greatly among genotypes. Quantification of soluble (A-C) and insoluble (D-F) concentrations of Aβ peptide in fly head extracts, analyzed using Meso Scale Discovery (MSD) immunoassay. Bars represent means ± SEM deduced from triplicate samples in three independent experiments.

In the soluble fraction, there were low amounts of Aβ in all genotypes, regardless of the C- or N-terminal truncations, varying between 0.5 and 4 ng/ml per fly (Fig 3A–3C). In the insoluble fractions, we observed a striking difference in Aβ levels between toxic and non-toxic peptides (Fig 3D–3F). Focusing first on the N-terminal variants, we observed similar levels of 3–42 and 1–42, with high insoluble amounts at both day 1 and day 10, and with soluble amount being relatively low at both time points (Fig 3B and 3E). Aβ levels in the 11–42 expressing flies were quite low considering their *in vivo* toxicity, ranging between 9–19 ng/ml insoluble Aβ. The 3–43 expressing flies had slightly lower Aβ levels when compared to the 1–43 expressing flies, but they displayed higher amounts when compared to the C-terminal truncated (1–37 to 1–41) peptides or the control (Fig 3C and 3F). At day 10, the ratio of insoluble/soluble Aβ was 3–4 times higher for the 11–42 expressing flies when compared to the 1–43 and 3–43 expressing flies, correlating with the more severe phenotype of 11–42 expressing flies (Fig 3B and 3E and S2 Table).

For C-terminal variants, we found that flies expressing the 1–42 peptide showed an insoluble amount of Aβ around 40 times higher than the soluble amount, varying between 56 ng/ml at day 1, to 86 ng/ml at day 10 (Fig 3A and 3D). A small increase in the insoluble level was also observed for the 1–43 expressing flies when compared to the C-terminal truncated peptides and the control (Fig 3C and 3F). This increase was augmented with age, ending at a soluble:insoluble ratio of 1:7. The overall ratio between soluble and insoluble Aβ in the C-terminal truncated peptides (1–37 to 1–41) was close to 1:1 and did not appreciably change over time (Fig 3D and S2 Table).

Taken together, these protein levels and solubility findings are in agreement with both the lifespan and activity assays, indicating that a reduced lifespan and impaired activity could be due to a high amount of accumulated insoluble Aβ.

### Histological analysis reveals correlation between aggregate load and toxicity

**Fig 4:**
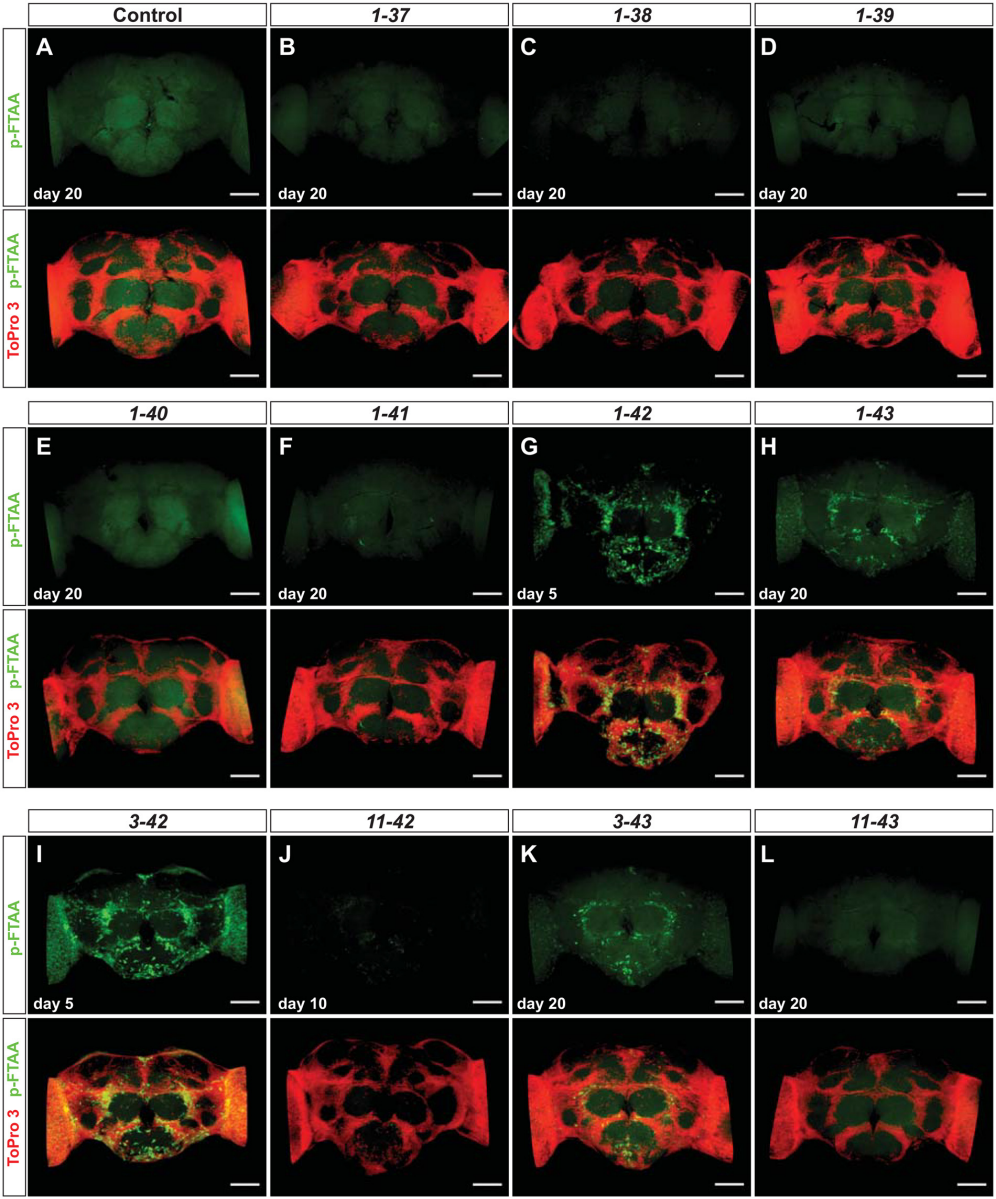
Aβ aggregation is in agreement with *in vivo* toxicity. (A-L) Whole *Drosophila* brain staining with the amyloid specific luminescent conjugated oligothiophene (LCO) p-FTAA (green) and the cell nuclei stain ToPro3 (red) at day 5, 10 or 20 (1–42 and 3–42 were not analyzed at day 20, due to their short lifespan). Scale bar = 50 μm. The results are representative for three independent experiments.

To address protein expression and aggregation in situ, we analyzed Aβ accumulation in whole brains using the protein aggregate-specific luminescent conjugated oligothiophene (LCO) p-FTAA [25]. In previous studies, using a whole brain staining protocol, we found co-staining between Aβ antibodies and p-FTAA in earlier fly models of AD [26]. For analyzing the improved fly model, flies were aged for 5, 10 or 20 days, depending on the genotype, before histological analyses were conducted. As expected, control flies did not display any punctate aggregate-like p-FTAA staining, although a diffuse background stain was observed over time (Fig 4A and S3 Fig). This background staining probably corresponds to a low level of endogenous aggregated proteins, becoming more abundant with age in flies. Similar results have been reported in *C*. *elegans*[27].

In the N-terminal truncated variants, we observed similar extent of aggregates in 3–42 expressing flies when compared to 1–42 expressing flies (Fig 4G vs 4I). Somewhat unexpectedly, flies expressing the 11–42 peptide displayed low amounts of aggregates, which contrasted to their rather severe life-span and locomotor phenotypes (Fig 4J). In comparison, the 1–43 or 3–43 expressing flies displayed similar, or even higher, extent of aggregates when compared to the 11–42 flies already at day 10 (not shown). Additionally, the 1–43 and 3-43-flies lived for about 10 days longer than the 11–42 flies, and at day 20 displayed more aggregates than the 11–42 flies at day 10 (Fig 4H, 4J and 4K). When analyzing the 11–43 flies, no aggregates were detected (Fig 4L).

In agreement with the lifespan and activity results, p-FTAA staining did not reveal any aggregates in flies expressing any of the four shortest C-terminal truncated variants (1–37, -38, -39, -40) at any time point (Fig 4A–4E). For the 1–41 expressing flies, a few punctate fibrillar structures could be seen at day 20 (Fig 4F). The p-FTAA fluorescence haze observed in the control flies could be observed in C-terminal truncated flies at day 20, and was most pronounced in 1–40 expressing flies (Fig 4A–4E). In contrast to these minor signals the p-FTAA staining revealed extensive Aβ aggregates and fibrillar structures in the 1–42 expressing flies, which were analyzed at day 5 due to their short life-span (Fig 4G). Moderate amounts of aggregates were also observed in the brains of flies expressing the 1–43 peptide at day 20 (Fig 4H) (aggregates were seen already at day 10, but the amount increased with age of the flies; not shown).

In all genotypes showing p-FTAA-positive aggregates, the location of the aggregates was very similar. Neither of these genotypes displayed aggregates with significant spectral differences from p-FTAA (data not shown), leading to the conclusion that the packing of the aggregates are as similar between genotypes as they are within a genotype, and thereby this should not be the main reason for the different toxicity observed.

### Mutating the N-terminus of 3–42 or 11–42 lends support for the pyro-glutamate toxicity model

**Fig 5:**
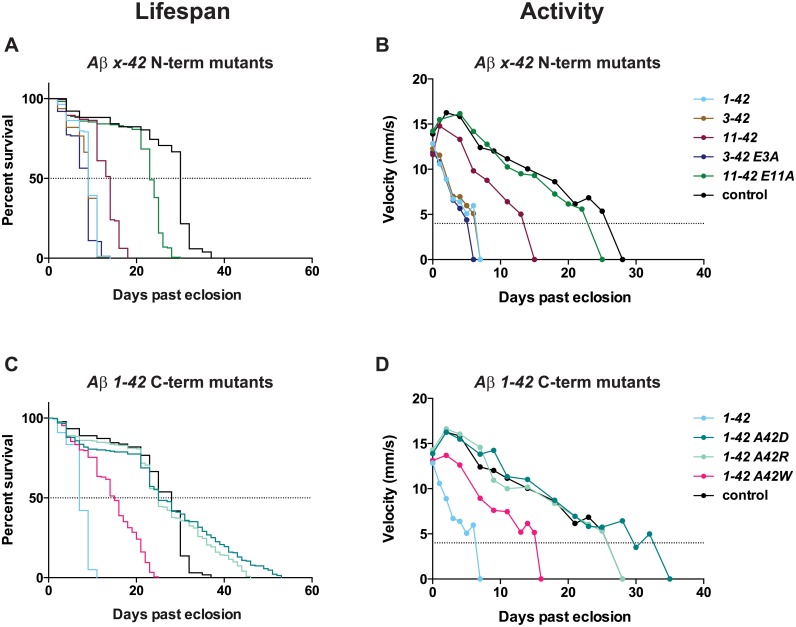
Expression of Aβ x-42 mutated at the N- or C-terminal improves lifespan and locomotor activity. Lifespan trajectories and locomotor behavior of *Drosophila* flies expressing Aβ in the nervous system. (A-B) N-terminal mutated peptides (3/11-42), (C-D) C-terminal mutated peptides ending at aa 42 (A42D/R/W). Survival plots were calculated using the Kaplan-Meier method. See S1 Table for statistical analyses of differences between the control and Aβ peptides.

**Fig 6:**
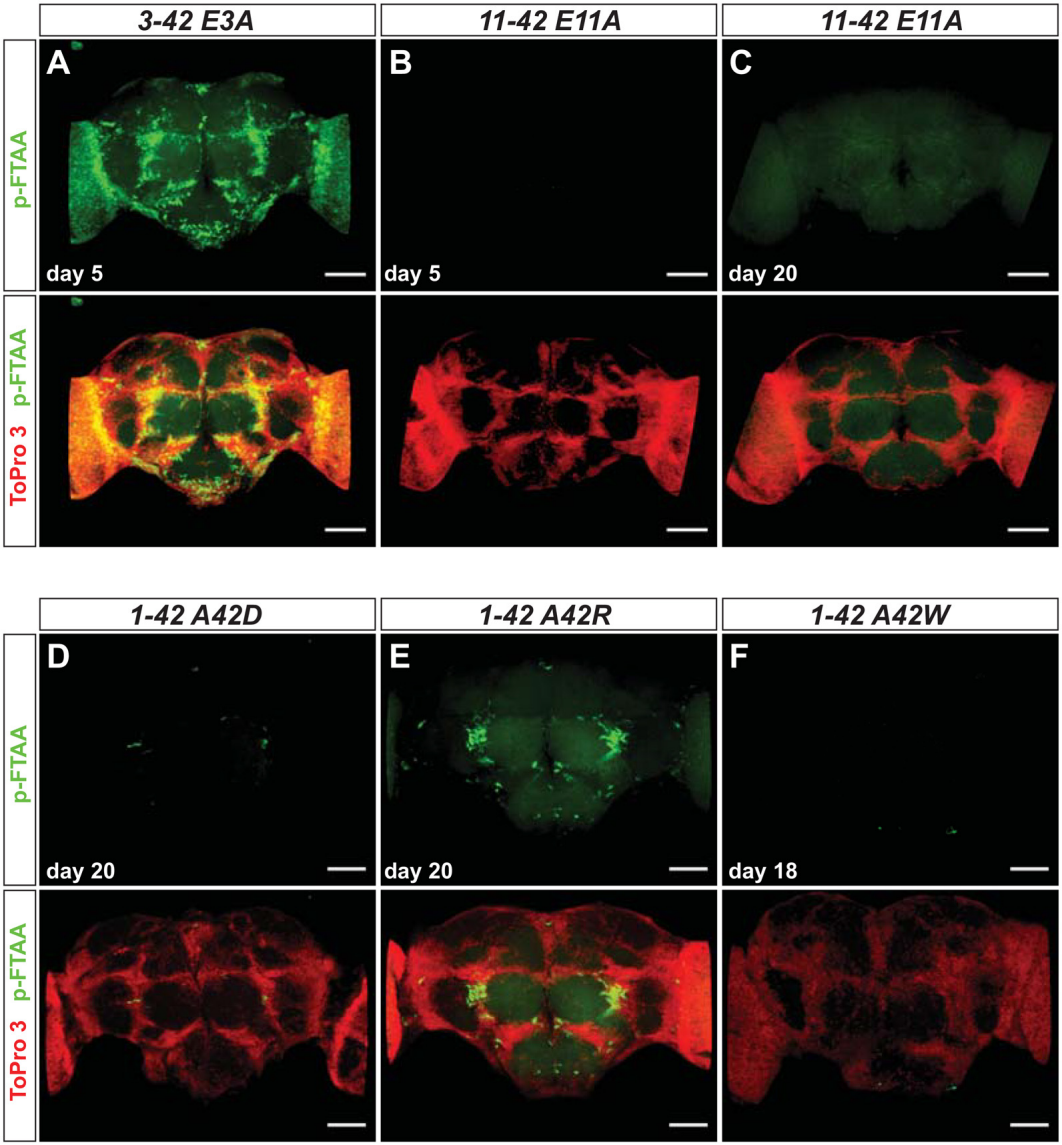
Protein aggregation is reduced in N- and C-terminally mutated Aβ variants. (A-F) Whole *Drosophila* brain staining with the amyloid specific luminescent conjugated oligothiophene (LCO) p-FTAA (green) and the cell nuclei stain ToPro3 (red) at day 5 or day 20. (A) The N-terminal mutated transgene *3–42 E3A* was not analyzed at day 20, due to its short lifespan. (B) C-terminal mutated transgene *1–42 A42W* was analyzed at day 18 due to its shorter lifespan. Scale bar = 50 μm. The results are representative for three independent experiments.

Previous studies have revealed a strong toxicity of N-terminal truncated variants with, in particular 3–42, but also 11–42, showing high toxicity [28]. Our results support this notion, with 3–42 similar in toxicity and aggregatory propensity to 1–42, and 11–42 also showing high toxicity, albeit less aggregatory propensity. The N-terminal aa residue of both 3–42 and 11–42 is a glutamate, and studies have pointed to a correlation between toxicity/aggregation and modification by cyclization of this residue into pyro-glutamate (pyroE)[29]. To address the putative toxicity of pyroE in the *Drosophila* system, we mutated both N-terminal residues of 3–42 and 11–42 (E3A and E11A; Fig 1) and addressed the effects thereof. Scoring toxicity and activity, we found no apparent change for E3A when compared to wild type, whereas E11A showed reduced effects (Fig 5A and 5B and S4A Fig). Protein analysis revealed partial reduction of both soluble and insoluble levels for both mutants (S1A and S1B Fig). In line with the toxicity and activity effects, the p-FTAA staining still revealed extensive aggregates in E3A, while E11A showed no clear aggregates (Fig 6A–6C). These results lend support for the importance of the N-terminal glutamate residue with respect to 11–42, and to some extent for 3–42 with respect to protein concentration (both soluble and insoluble).

To address the possible presence of pyroE modification of 3–42 in *Drosophila*, we used an Aβ 3–42 pyroE-specific antibody combined with the 4G8 Aβ antibody, and analyzed protein content using the MSD immune-assay. This revealed that while we could not detect Aβ 3–42 pyroE in the soluble fraction, at neither 1 nor 10 days of age, pyroE was indeed detected in the insoluble fraction, at day 10 (S6 Fig). Aβ 3–42 pyroE composed a minor fraction (around 20%) of the total 3–42 found in the insoluble fraction. In line with the specificity of the antibodies, pyroE was below detection in fly extracts from both the 3-42E3A and 1–42 flies, as well as on synthetic 1–42 peptide (not shown). We were not able to establish an MSD immune-assay for Aβ 11–42 pyroE (see Materials and Methods).

### Mutating the C-terminus of 1–42 results in striking reduction of toxicity

The striking difference in toxicity between 1–42 when compared to a C-terminal extension (1–43) or several truncations (1–41, -40, -39, -38, -37) pointed to the critical role of position 42. To address this residue further, we generated three different mutations: A42D, A42R and A42W. Surprisingly, all three mutations led to clear reduction in toxicity and increased performance in the activity assay (Fig 5C and 5D and S4B Fig). These three mutants showed significant differences in toxicity when compared to each other, with W more toxic than R, which was more toxic than D (W>R>D; S1B Table). Additionally, although soluble protein levels were not vastly different, all three mutations displayed strong reduction of insoluble protein amounts when compared to wild type (S1C and S1D Fig). The reduced toxicity *in vivo* and reduced insoluble protein aggregation was furthermore mirrored by reduced p-FTAA staining, and only 1–42 A42R displayed clear aggregate staining (Fig 6D–6F). Interestingly, this was not in complete agreement with the observed toxicity (W>R>D). In summary, these results underscore the importance of aa residue 42, and also points to the specific importance of an alanine amino acid for high toxicity and aggregatory propensities.

## Discussion

**Fig 7:**
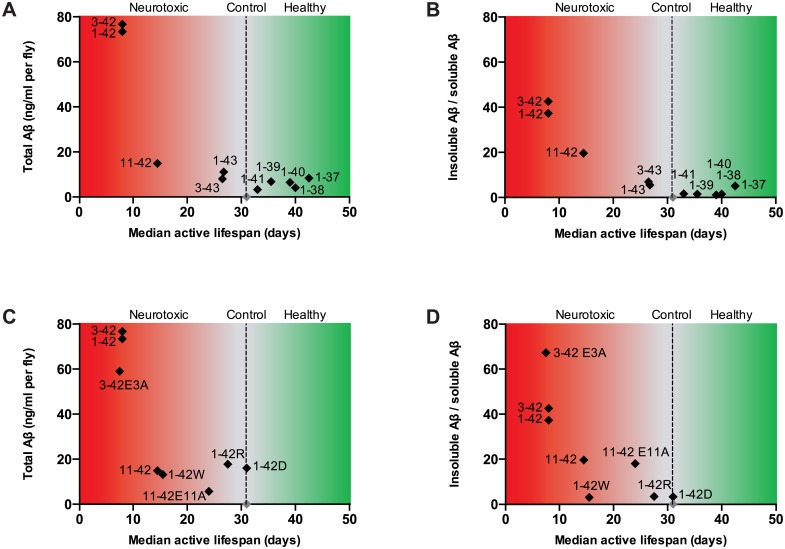
Median active lifespan versus accumulation of Aβ. Median lifespan plotted versus total Aβ accumulation (ng/ml per fly) (A and C) or versus insoluble/soluble Aβ (B and D). (A) and (B) showing peptides expressing various lengths of the Aβ peptide, (C) and (D) showing peptides that are mutated in either the N- or C-terminal of the Aβ peptide. Median active lifespan is; (the median lifespan + the day were the activity reaches the cut-off value) / 2.

A major focus of research into AD has been on the amyloidogenicity of Aβ 1–42. However, due to the sequential cleavage of APP and subsequent processing of Aβ, a pool of various Aβ peptides is generated. While several studies have considered the aggregation properties of different peptides [9, 23, 30, 31] the importance of the various peptides *in vivo* has not been systematically addressed. Widely studied transgenic mouse models of AD, Tg2576, APP23, APP^swe^ are over-expressing APP with a Swedish mutation [32, 33], hence affording limited control of the resulting Aβ peptides following β- and γ-secretase cleavage and proteolytic processing. With double transgenics e.g., APP/PS1 [34] additional control over the Aβ 40/42 ratios could be differentiated, albeit still mixed and with the presence of additional peptides of unclear importance. Moreover, although mouse studies have been conducted that over-express the Aβ peptide directly in the absence of APP, so far only the Aβ 1–40, 1–42 and 3–42 peptides have been investigated [35, 36]. Previous studies expressing Aβ in *Drosophila* have, to our knowledge, focused on the expression of the 1–40 and 1–42 peptides [11–13, 18, 21]. In the present study we aimed to address if different Aβ peptides may differentially contribute to the Aβ toxicity observed in Alzheimer’s disease by the generation of 16 different *UAS* transgenic *Drosophila* strains. To facilitate these studies, we improved the *Drosophila* model by several changes, including an improved *Gal4* driver line, improved *UAS-Aβ* transgenes and landing site transgenesis. Taken together, the results for the various genotypes in this study are in general agreement; decrease in lifespan and locomotor activity parallels increase in Aβ accumulation and total Aβ content and aggregation (Fig 7A–7D and S7 Fig).

### N-terminal effects: The importance of E3 and E11

N-terminally modified Aβ peptides have been reported, with the most prominent forms identified starting at position 3 or 11 and possessing N-terminal pyroglutamic acid (pyroE), generated from glutamic acid [37]. Previous *in vitro* studies have shown that N-terminal deletions accelerate Aβ aggregation into neurotoxic fibrils [38, 39]. To address N-terminal toxicity in the fly model, we generated four N-terminal truncated variants, two starting at aa 3 and two at aa 11. In our *Drosophila* models we did not observe a clear difference in lifespan, activity or Aβ accumulation between the 1-42/43 and 3-42/43 expressing flies. In the case of 1–42 and 3–42 expressing flies, one reason for their very similar phenotype could be that expression of the 1–42 peptide is highly toxic in itself, and hence a minor increase in toxicity by the 3–42 peptide in flies might be difficult to distinguish. When expressing the 11–42 variant, we observed increase in lifespan and activity, and a significant decrease in Aβ accumulation in fly brains when compared to the longer peptides 1–42 and 3–42. Notwithstanding, 11–42 is still highly toxic to flies when compared to the majority of the Aβ peptides tested herein. This result again highlights the crucial importance for residue 42 in mediating neurotoxicity. The mitigated toxicity effect was also seen for the 11–43 expressing flies, whereas the moderate toxicity seen in 1–43 and 3–43 expressing flies was abolished. To address the importance of the E3 or E11 residues, we mutated them to alanine. This did not apparently affect 3–42 toxicity, while E11A was less toxic compared to 11–42, lending support for the possibility of pyroE toxicity at residue 11. However, the E3A mutant did display reduced both soluble and insoluble protein, lending some support for the toxicity also linked to E3, likely by reduced degradation. The elevated insoluble/soluble ratio for E3A (Fig 7D) implies that removal of a charged E residue can influence the solubility. However, this effect is complicated to interpret, because pyroE formation removes two changes (N-terminal and E side-chain), and in addition, it is unlikely that all peptides are simultaneously cyclized to pyroE. Indeed, while our protein analysis of pyroE revealed the presence of pyroE, this modification was only detected in some 20% of the 3–42 protein present in the insoluble fraction. Previous studies have identified two glutaminyl cyclases in *Drosophila* [40], and our identification of pyroE on 3–42 provides in vivo support for their activity. Mutating the genes encoding the *Drosophila* enzymes and/or introducing the human enzyme transgenically may help shed further light on pyroE Aβ toxicity in the fly model.

For 11–42 expressing flies the effect was mostly evident from accumulation of insoluble versus soluble Aβ, which either appears to reach a threshold, or more likely reveal the crucial neurotoxic effect of A42. These flies show a relatively short lifespan, with impaired activity, and moderate total protein levels, but they do not accumulate p-FTAA positive aggregates to the same degree that their phenotype may suggest. This is evident especially when comparing these flies to the 1–43 or 3–43 expressing flies. At day 10, which is about 40% of the total lifespan of the 1–43 and 3–43 flies and 70% of the 11–42 expressing flies, 11–42 expressing flies reveal some 3–4 times higher insoluble/soluble Aβ ratio, when compared to 1–43 and 3–43 expressing flies (Fig 7B). Despite that, whole brain staining revealed similar, or even higher, amounts of visible aggregates in the 1–43 and 3–43 expressing flies when compared to 11–42, indicating that the amount of large aggregates is not enough to induce toxicity in flies, at least not at moderate levels of aggregates. These data are in agreement with previous findings from our group when analyzing the 1–42 (E22G) Arctic-mutation with previous *Drosophila* models. We ascribed the superior neurotoxicity of the Arctic mutation to specific oligomerization rather than histologically detected mature fibrillar aggregates [13]. In addition, feeding curcumin, which hastened the maturation of pre-fibrillar oligomeric aggregates towards mature fibrils, was hereby neuroprotective especially for these flies [13], without changing the total amount of Aβ. In the case of 1–42 and 3–42 expressing flies, the high amounts of insoluble Aβ and the amount of aggregates are probably challenging the degradation systems in the nervous system of the fly. The role of 11–42 in AD pathogeneses is unclear, but it has been suggested that this peptide has an early role in Aβ deposition into plaques [6].

### C-terminal effects: The importance of Ala 42

The combined result from the lifespan and activity assays show that while 1–42 is highly toxic, expressing C-terminally truncated peptides shorter than 42 has no toxic effect. These C-term truncations result in very low amounts of total Aβ, and do not reveal amyloid staining with p-FTAA. Since we have the same chromosomal insertion of each Aβ construct, and hence have negated any transgenic insertion positional effect, the lack of toxicity seen in these flies is probably due to decreased aggregation and/or increased degradation, which is thought to be a crucial patho-mechanism in AD [41]. In stark contrast to this, flies expressing the 1–42 peptide display strong phenotype, with severely reduced lifespan, locomotor dysfunction, extensive p-FTAA-positive aggregates and high amounts of insoluble protein (Fig 7A–7D). The 1–42 peptide is well-known to be more aggregation prone than other peptides *in vivo*[14, 42], and as a consequence the degradation system is apparently unable to cope with the high amount of misfolded protein accumulated in our transgenic flies. Extending the peptide beyond 1–42, several studies have focused on the 1–43 peptide, both *in vitro* and *in vivo*, showing it to be of importance for AD [43–45]. Mouse studies indicate that 1–43 is potently amyloidogenic and pathogenic *in vivo*, and that Aβ 1–43 appears to be as prone to aggregate *in vitro* as 1–42 [7, 23]. These findings are rather contradictory to our results, were we see distinct difference in toxicity between the 1–42 and 1–43 expressing flies. 1–43 expressing flies do show decreased lifespan and activity when compared to controls, and appearance of p-FTAA positive aggregates, but these effects are much less severe than those observed in the 1–42 expressing flies (Fig 7A and 7B). Moreover, our results are in agreement with a recent study comparing 1–42 with 1–43, showing that 1–42 is more toxic than 1–43, with respect to life-span, locomotion, neurodegeneration and protein aggregation [46]. The C-terminal aa residues of 1–42 are isoleucine and alanine, which increases the hydrophobicity of the peptide when compared to shorter variants. Position 43 is a threonine, which carries a hydrophilic OH-group, and this may be one of the reasons for the reduced aggregation and lower toxicity observed in these flies when compared to the 1–42 expressing flies. These results strongly implicate specific folding and oligomerization of 1–42 being hindered by the 43 extension. To further explore this hypothesis we generated flies expressing 1–42 with the synthetic mutations A42D, A42R and A42W. This resulted in a striking loss of toxicity for A42D or A42R flies (Fig 5C), while A42W showed an intermediate toxicity when compared to 1–42. The stringent requirement of the A42 residue fits with several structure models and biochemical data concerning 1–42 aggregation and fibrillation. Aβ fibrils display vast structural polymorphism, notwithstanding there are several different models for synthetic 1–42 and 1–40 amyloid fibrils [47–51]. In general (despite polymorphism) the models agree on a hidden C-terminal sequence (30-40/42) and central region (15–25), with a partially accessible N-terminal region encompassing the initial 10–12 residues. The hidden regions make up an in-register cross-β-sheet formation, with a bend around residues 25–29 to generate a folded protomer contacting the central region and with the C-terminal sequence allowing a dry hydrophobic core to form. Intermolecular hydrogen bonds form between the β-strands running perpendicular to the fibril axis. An extension of the core by two additional hydrophobic residues (Ile-Ala) in 1–42 is a generally accepted view of increased aggregation of this peptide compared to 1–40, and likely also has effects of further annealing of the central β-sheet [49]. Interestingly the structural basis for specificity of Aβ aggregation behavior have somewhat counterintuitively implicated that the mere hydrophobicity is driving its aggregatory state. A random substitution of twelve C-terminal residues of 1–42 still aggregated, suggesting a high promiscuity of the interactions as the driving force for 1–42 aggregation [52]. In contrast, our results point to a highly sequence-specific effect for mediating *in vivo* neurotoxicity. Specific oligomerization of 1–42 rather than fibril formation has been discussed along these lines initiated by a highly influential paper by Klein [52]. As a molecular explanation for this observation it has been reported that 1–40 forms monomers, dimers, trimers and tetramers in rapid equilibrium whereas 1–42 forms “paranuclei” *i*.*e*. pentameric or hexameric subunits with the ability to grow towards protofibrils [53]. Importantly N-terminal truncations did not affect 1–42 “paranuclei” formation [54]. However, this behavior of 1–42 was virtually identical for 1–43 and was dictated by residue 41; residue A42 was necessary for further polymerization. Specifically, A42 was also significant for further polymerization of the “paranuclei” as revealed by an A42G substitution [54]. Our *in vivo* results now point to that 1–43 is much less toxic than 1–42, and that substitutions at position 42 (A42D/R/W) greatly reduces toxicity and aggregation; together demonstrating crucial importance of A42. Furthermore, our results *in vivo* are difficult to explicitly correlate to fibrillar models, because all structures investigated do fibrillate *in vitro* (1–37–1–43), albeit with different kinetics. Of note, we also find aggregates in A42R, while this variant shows rather mild phenotype in terms of neurotoxicity. Interestingly, all our results around the C-terminus are compatible with a recently proposed symmetric hexameric β-barrel oligomer model by the Härd group [55]. Herein I41 and A42 are hidden in a domain-swapped structure, where a C-terminal β-strand (residues 39–42) is paired with an antiparallel β-strand (residues 34–36) in an adjacent protomer. This model is highly compatible with our observations that single C-terminal residue changes (extension, truncation and substitution) directly impose structural constraints that preclude such a specific fold to form. Because flies expressing the most neurotoxic peptides show exceptional accumulation of insoluble and p-FTAA positive aggregates, our results correlate well with a recent proposal of a dynamic interplay of monomeric, oligomeric and protofibrillar 1–42 i.e., the actual process of fibrillation and oligomerization is the driving force behind neurotoxicity [56].

In summary, our study demonstrates that Aβ peptides ending at aa 42 shows high neurotoxicity in *Drosophila* (Table 1). Rather unexpectedly, this deleterious effect can be completely or mostly abolished by either removal or addition of a single C-terminal aa. Strikingly, we find that the C-terminal residue A42 is itself critical, since mutating the A to D, R or W, resulted in strongly reduced toxicity. In contrast to previous studies of the 1–43 peptide [7], we found that flies expressing this peptide were only moderately affected when compared to the control flies. Mutagenesis of E>A for 3–42 and 11–42 furthermore lent support for the pyroE notion, in particular for 11–42. The striking effects of x-42 peptides were linked to two specific features: a) high neurotoxicity and b) limited degradation. Due to the elevated aggregation propensity of x-42 peptides, our results highlight the efficiency of rapid oligomerization of these peptides to form specific stable neurotoxins, and supports the strategy aimed at decreased formation of x-42 peptides as a relevant therapeutic strategy.

**Table 1:** Summary of lifespan, activity, aggregate load in adult brain and protein concentration.

Genotype	Lifespan	iFly	Aβ aggregates d5	Aβ aggregates d10	Aβ aggregates d20	Conc. Aβ d1	Conc. Aβ d10	Conc. Aβ d20
***1–37***	**-**	**-**	**n/a**	**-**	**-**	**-**	**-**	**-**
***1–38***	**-**	**-**	**n/a**	**-**	**-**	**-**	**-**	**-**
***1–39***	**-**	**-**	**n/a**	**-**	**-**	**-**	**-**	**-**
***1–40***	**-**	**-**	**n/a**	**-**	**-**	**-**	**-**	**-**
***1–41***	**-**	**-**	**n/a**	**-**	**-**	**-**	**-**	**-**
***1–42***	**✝✝✝**	**✝✝✝**	**+++**	**n/a**	**d**	**++**	**+++**	**d**
***1–43***	**✝**	**✝**	**n/a**	**++**	**++**	**-**	**+**	**+**
***3–42***	**✝✝✝**	**✝✝✝**	**+++**	**n/a**	**d**	**++**	**+++**	**d**
***11–42***	**✝✝**	**✝✝**	**+**	**+**	**d**	**+**	**+**	**d**
***3–43***	**✝**	**✝**	**n/a**	**++**	**++**	**-**	**+**	**+**
***11–43***	**-**	**-**	**n/a**	**-**	**-**	**-**	**-**	**-**
***3–42 E3A***	**✝✝✝**	**✝✝✝**	**+++**	**n/a**	**d**	**++**	**+++**	**d**
***11–42 E11A***	**✝**	**✝**	**-**	**-**	**-**	**-**	**-**	**-**
***1–42 A42D***	**✝**	**-**	**-**	**+**	**+**	**+**	**+**	**+**
***1–42 A42R***	**✝**	**-**	**+**	**++**	**++**	**+**	**+**	**+**
***1–42 A42W***	**✝✝**	**✝**	**-**	**-**	**-**	**+**	**+**	**+**
***Oregon-R***	**-**	**-**	**-**	**-**	**-**	**-**	**-**	**-**

Lifespan and activity were categorized as (-) = no effect; (✝) = mild effect; (✝✝) = strong effect; (✝✝✝) = very strong effect. Aβ histo-aggregates refers to amount of p-FTAA aggregates detected in adult brains, and were categorized as (-) = no aggregates; (+) = some aggregates; (++) = extensive aggregates; (+++) = very extensive aggregates. Conc. Aβ refers to protein concentration and was categorized as (-) = low; (+) = intermediate; (++) = high; (+++) = very high.

## Materials and Methods

### *Drosophila* stocks

*C155-elav-Gal4* and *n-syb-Gal4* (Bloomington Stock Center #458 and #39171, respectively). *UAS-nls-myc-eGFP* (obtained from D. van Meyel; denoted *UAS-eGFP* in S1 Fig). *n-syb-Gal4#2–1*, original insert on 3rd chromosome (kindly provided by Julie Simpson). As control, the *n-syb-Gal4* was crossed to *Oregon-R* flies.

### Transgenics

The sequence coding for human Aβ was codon optimized for expression in *Drosophila* (www.kazusa.or.jp/codon/cgi-bin/showcodon.cgi?species). Sequences were added to the 5’: a consensus start codon [57] and an EcoRI site, as well as to the 3’; three different stop codons (amb, och, opa) and an XbaI site (see S1 Suppl. Info. for all sequences). For the signal sequence, we ran the two signal sequences previously used to express Aβ, taken from the pre-proEnkephalin and Necrotic proteins [11, 18, 21], against two different signal sequence prediction databases: SignalP 4.0 (http://www.cbs.dtu.dk/services/SignalP/) and SIG-Pred (http://bmbpcu36.leeds.ac.uk/prot_analysis/Signal.html). Both sites predicted that the pre-proEnkephalin sequence would be the most efficiently cleaved, and most consistently cleaved at the same aa position, of the two sequences, and we thus used this signal sequence. DNAs were generated by gene-synthesis (Genscript, New Jersey, USA), and cloned into pUASattB [16], as EcoRI/XbaI fragments. DNAs were control-sequenced on both strands in the UAS vector (GATC BioTech, Germany), and injected into landing site strains BL#9750; 65B and BL#9744; 89E (BestGene, CA, USA). *n-syb-Gal4*, a *P* element construct (kindly provided by Julie Simpson), was injected into *y*,*w*, and 62 new transgenes were generated (BestGene Inc., CA, USA). These were tested against *UAS-eGFP* for expression levels. High expressers, on the 2^nd^ and 3^rd^ chromosome, were combined by recombination. For this study, a double transgenic line, *n-syb-Gal4#2–1*, *1M* was mostly used.

### Lifespan assay

Flies were kept at 60% humidity at +25°C under a 12:12 h light:dark cycle until eclosion and at +29°C post eclosion. The crossings were reared in 50 ml vials containing standard *Drosophila* food (corn meal, molasses, yeast and agar). Newly eclosed flies, corresponding to the *n-syb-Gal4* crossings, were maintained at +29°C in 50 ml vials (20 flies per vial) containing rich *Drosophila* food (water, potato mash powder, corn flour, yeast, agar, syrup, propionic acid (diluted: 48.5 ml propionic acid + ~950 ml H_2_O) and green food coloring). Every 2–3 days the flies were transferred to fresh vials and the number of surviving flies was recorded throughout the lifetime of all flies. The assay was repeated three-five times and a total of between 106–244 flies per genotype were assayed. The data was pooled and analyzed together. GraphPad Prism 6.0a software (GrapPad Software Inc., San Diego, CA, USA) was used to generate Kaplan-Meier survival curves [58] and to run the log-rank statistical analysis. The definition of significance was p-values of less than 0.05 (*), 0.01 (**), 0.001 (***) and 0.0001 (****).

### Activity assay

The locomotor behavior of individual flies was recorded using a locomotor assay, iFly [24]. For each genotype three vials with 10 newly eclosed flies in each vial were assayed from a total of five vials. A movie of 90 s was recorded for each vial, and every 30 s the flies were tapped to the bottom of the vial to ensure the same starting point in each movie, yielding nine movies of 30 s for each genotype and time point. Movies were recorded every 2–3 days until the flies were to immobile to be able to record. To follow the recommendations for the iFly system we used 10 flies for each 90 s movie. Hence, the three assay vials were replenished to compensate for dead flies during aging, which otherwise could have resulted in a selection bias for viable flies. The movies were processed using the iFly software and the parameters velocity and angel of movement were calculated. The data was analyzed using GraphPad Prism 6.0a software (GraphPad Software Inc., San Diego, CA, USA).

### Quantification of the Aβ-peptide levels

#### Sample preparation

Flies expressing the Aβ 1–42, 3–42, 3–42 E3A and 11–42 peptides were aged to 1 or 10 days, due to their short lifespan, while the remaining genotypes were aged to 1, 10 or 20 days. Samples were prepared as described in [14], with the exception that flies corresponding to the Aβ expressing flies ending at amino acid 42 were diluted 5 times more than the other flies prior to addition to the plate due to their high levels of Aβ. All samples were stored at -80°C until use.

#### Immuno-assay

The quantification of Aβ peptides in the “soluble” and “insoluble” fractions were performed using a standard binding MSD 96-Well MULTI-ARRAY plate (L15XA-3, Meso Scale Discovery, MD, USA). The plate was coated with 25 μl, 10 μg/ml of either a 6E10 monoclonal antibody (SIG- 39320, Nordic Biosite, Sweden) or a 12F4 monoclonal antibody (an anti-Aβ42 ab) (SIG- 39142, Nordic Biosite, Sweden) depending on the Aβ peptide to be analyzed (1h, RT, with gentle agitation). For capturing the 3–42 or 11–42 pyroE modifications, Pyro Glu3 and Pyro Glu11 antibodies (Novus Biologicals, Abingdon, UK; Cat#: NBP1-44048 and NBP1-44070) were used at 10–20 μg/ml. As calibrators, [Pyr3]-β-Amyloid (3–42) or [Pyr11]-β-Amyloid (11–42) peptides (Cat#: AS-29903-01 and AS-29907-01, AnaSpec, USA) was used, ranging from 0–10 000 pg/ml. This concentration range was the same as for the Aβ 1–42 peptide calibrator (C01LB-2, Meso Scale Discovery, MD, USA). pyroE was below detection in head extracts from both the 3-42E3A and 1–42 flies, as well as on synthetic 1–42 peptide (not shown). While the assay for 3–42 pyroE was successful, detection of the 11–42 pyroE peptide did not work in our hands. The plate was washed three times with 150 μl 1x Tris Wash Buffer (R61TX-2, Meso Scale Discovery, MD, USA) and blocked with 150 μl/well 1% MSD Blocker A solution (R93BA-1, Meso Scale Discovery, MD, USA) (30 min, RT, agitation). Triplicate 25 μl aliquots of sample were mixed with an equal amount of MSD Blocker A (2% MSD Blocker A, 0.2% Tween 20 and protease inhibitor) and added to the plate (1h, RT, gentle agitation). The plate was washed and detection was achieved by addition of 25 μl 1x SULFO-TAG—conjugated 4G8 detection antibody (D20RQ-3, Meso Scale Discovery, MD, USA) (1h, RT, gentle agitation). The plate was once again washed and 150 μl 2X read buffer (R92TC-2, Meso Scale Discovery, MD, USA) was added to the plate. Measurements were taken in a SECTOR Imager 2400 instrument (Meso Scale Discovery, MD, USA). To adjust for variation in the protein extraction step a quantitation of the total amount of protein from each sample of fly homogenate was performed by usage of the Bio-Rad DC Protein Assay Kit II (500–0112; BioRad, CA, USA).

### Histological analysis

Whole *Drosophila* brains were assayed by histological staining for the presence of amyloid deposits using the amyloid specific luminescent conjugated oligothiophene (LCO), p-FTAA [25]. Female flies corresponding to the *n-syb-Gal4* crossings were reared at +29°C until 5, 10 or 20 days after eclosure. A silicon rubber well was made on a poly-lysine adhesive slide (Fisher Scientific) and decapitated fly heads were dissected in PBS, using two pairs of fine forceps, and placed on the slides prior to staining. Brains were fixed in 96% ethanol for 10 minutes and re-hydrated to distilled water in 2-min steps, in 70%, 50% and 0% ethanol at room temperature. Slides were washed in PBS (5 min, RT) prior to addition of 3μM p-FTAA diluted in PBS (30 min, RT). After incubation with p-FTAA, slides were washed in PBS (3x5 min, RT). To visualize cell nuclei, brains were stained with 5 μM ToPro3 (TO-PRO-3; Life technologies) diluted in PBS (15 min, RT). Slides were once again washed in PBS (5 min, RT) and rinsed in distilled water (2x5 min, RT). The silicon rubber well was removed and two cover slips were aligned on each side of the dissected *Drosophila* brains to produce a spacer. Nail polish was used to attach the coverslips to the slide. Slides were allowed to dry at room temperature, mounted in DAKO mounting medium (DAKO #S3023; DAKO, Glostrup, Denmark), and stored at +4°C overnight. All incubations were carried out in a dark chamber to minimize the risk of bleaching. Prior to imaging, the slides were sealed with nail polish. For each genotype, a minimum of three brains was analyzed. A Zeiss LSM 780 confocal microscope was used for fluorescent images; confocal stacks were merged and processed using LSM software or Adobe Photoshop. All images were processed using the same procedure. Micrographs were analyzed for p-FTAA positive aggregates, and an estimation of the aggregate load in each genotype was done by visual inspection in the fluorescence microscope. Images and graphs were compiled in Adobe Illustrator.

Analysis of eGFP expression in the brain was performed by dissections as outlined above, and the eGFP signal was collected on a Zeiss LSM510 META system. eGFP protein levels in fly head extracts was determined using Western blot and ECL detection, with an antibody against GFP at 1:1,000 (Milllipore, MA, USA).

## Supporting Information

S1 Fig Generation of a stronger *Gal4* line. (A-L) Expression of eGFP in dissected adult fly brains. (M) Expression of eGFP on western blot, and (N) quantification (α-tubulin was used as an internal standard). Using both methods, the multi-insert *nsyb-Gal4* driver gives stronger eGFP expression when compared to the *C155-elav-Gal4* line, particularly in females, and increasingly so with age. (O) Comparison of life-span of flies expressing Aβ 1–42 driven from the multi-insert *n-syb-Gal4* used in this study and the *n-syb-Gal4* stock available at Bloomington Stock Center (#39171). The experiment was conducted at +20°C. The multi-insert driver shows significantly shorter life-span.
(EPS)

S2 Fig Locomotor behavior of flies expressing Aβ is in agreement with lifespan. Locomotor behavior of flies expressing different Aβ peptides, analyzed by angle-of-movement. The cut-off value is when the angle reaches 80°.
(EPS)

S3 Fig Aging of flies increase overall protein aggregation. Whole *Drosophila* brain staining of *n-syb-Gal4/Or-R* control with the amyloid specific luminescent conjugated oligothiophene (LCO) p-FTAA (green) and the cell nuclei stain ToPro3 (red) at day 5 or day 20.
(EPS)

S4 Fig Locomotor behavior of flies expressing mutated Aβ peptides is in agreement with lifespan. Locomotor behavior of flies expressing Aβ peptides mutated at the N- or C-terminal, analyzed by the angle-of-movement. The cut-off value is when the angle reaches 80°.
(EPS)

S5 Fig Effects of soluble and insoluble protein level in N- or C-terminal mutated Aβ peptides. Quantification of soluble (A, C) and insoluble (B, D) concentrations of Aβ peptide in aged flies, performed by the Meso Scale Discovery (MSD) immunoassay. Bars represent means ± SEM. deduced from triplicate samples in three independent experiments.
(EPS)

S6 Fig pyroE levels in 3–42 flies. Quantification of soluble (A) and insoluble (B) concentrations of Aβ and Aβ pyroE peptides in aged flies, performed by the Meso Scale Discovery (MSD) immunoassay. pyroE-modified Aβ is detected at 10 days of age, specifically in the 3–42 flies. pyroE was below detection in the 3-42E3A mutant (not shown). Bars represent means ± SEM, pooled from two independent assays.
(EPS)

S7 Fig Median lifespan and activity versus accumulation of Aβ. Median lifespan and locomotor velocity plotted versus soluble and insoluble Aβ accumulation (ng/ml per fly). (A-D) wild type proteins; (E-H) mutated proteins.
(EPS)

S1 Supplemental Information Contains DNA sequences for the different Aβ transgenic constructs generated in this study. (PDF)

S1 Table Median lifespan of different Aβ transgenes. (A) Median lifespan for different Aβ transgenes, including N-terminal aa mutants, total number of flies assayed, number of independent lifespan assays and significance versus Oregon-R (control). (B) Equivalent data for C-terminal aa mutations.
(PDF)

S2 Table Comparison of mean Aβ protein concentration in soluble and insoluble fraction. Concentration of soluble and insoluble Aβ in fly head extracts, as measured by the Meso Scale Discovery (MSD) immunoassay. n/a = not assayed; d = dead at time-point.
(PDF)
