# Supplementary material for: Systematic Aβ Analysis in Drosophila Reveals High Toxicity for the 1-42, 3-42 and 11-42 Peptides, and Emphasizes N- and C-Terminal Residues
Source: PLoS One. 2015 Jul 24;10(7):e0133272. doi: 10.1371/journal.pone.0133272 (PMC4514787; doi:10.1371/journal.pone.0133272)
Supplement: S1 Supplemental Information — (PDF) [file pone.0133272.s008.pdf]

Supplemental Information 1

Synthetic Aβ DNA sequences

EcoRI and start codon (bold underlined), stop codons (blue, bold, underlined), XbaI (green, bold, underlined), mutated codons (red, bold, underlined).

1-42
gaattcaaccaaa**atg**gcccagttcctgcgcctgtgcatctggctgctggccctgggtagctgcctgctggccaccgtgcaggccgatg
ccgagttccgccacgatagcggttacgaggtgcaccaccagaagctggtgttcttcgccgaggatgtgggtagcaacaagggtgccatc
atcggtctgatggtgggtggtgtggtgatcgcc**taatagtgatctaga**

1-41
gaattcaaccaaa**atg**gcccagttcctgcgcctgtgcatctggctgctggccctgggtagctgcctgctggccaccgtgcaggccgatg
ccgagttccgccacgatagcggttacgaggtgcaccaccagaagctggtgttcttcgccgaggatgtgggtagcaacaagggtgccatc
atcggtctgatggtgggtggtgtggtgatc**taatagtgatctaga**

1-40
gaattcaaccaaa**atg**gcccagttcctgcgcctgtgcatctggctgctggccctgggtagctgcctgctggccaccgtgcaggccgatg
ccgagttccgccacgatagcggttacgaggtgcaccaccagaagctggtgttcttcgccgaggatgtgggtagcaacaagggtgccatc
atcggtctgatggtgggtggtgtggtg**taatagtgatctaga**

1-39
gaattcaaccaaa**atg**gcccagttcctgcgcctgtgcatctggctgctggccctgggtagctgcctgctggccaccgtgcaggccgatg
ccgagttccgccacgatagcggttacgaggtgcaccaccagaagctggtgttcttcgccgaggatgtgggtagcaacaagggtgccatc
atcggtctgatggtgggtggtgtg**taatagtgatctaga**

1-38
gaattcaaccaaa**atg**gcccagttcctgcgcctgtgcatctggctgctggccctgggtagctgcctgctggccaccgtgcaggccgatg
ccgagttccgccacgatagcggttacgaggtgcaccaccagaagctggtgttcttcgccgaggatgtgggtagcaacaagggtgccatc
atcggtctgatggtgggtggt**taatagtgatctaga**

1-37
gaattcaaccaaa**atg**gcccagttcctgcgcctgtgcatctggctgctggccctgggtagctgcctgctggccaccgtgcaggccgatg
ccgagttccgccacgatagcggttacgaggtgcaccaccagaagctggtgttcttcgccgaggatgtgggtagcaacaagggtgccatc
atcggtctgatggtgggt**taatagtgatctaga**

3-42
gaattcaaccaaa**atg**gcccagttcctgcgcctgtgcatctggctgctggccctgggtagctgcctgctggccaccgtgcaggccgagt
tccgccacgatagcggttacgaggtgcaccaccagaagctggtgttcttcgccgaggatgtgggtagcaacaagggtgccatcatcggt
ctgatggtgggtggtgtggtgatcgcc**taatagtgatctaga**

11-42

gaattcaaccaaa**atg**gcccagttcctgcgcctgtgcatctggctgctggccctgggtagctgcctgctggccaccgtgcaggccgag
gtgcaccaccagaagctggtgttcttcgccgaggatgtgggtagcaacaagggtgccatcatcggtctgatggtgggtggtgtggtgatc
gcc**taatagtgatctaga**

1-43
gaattcaaccaaa**atg**gcccagttcctgcgcctgtgcatctggctgctggccctgggtagctgcctgctggccaccgtgcaggccgatg
ccgagttccgccacgatagcggttacgaggtgcaccaccagaagctggtgttcttcgccgaggatgtgggtagcaacaagggtgccatc
atcggtctgatggtgggtggtgtggtgatcgccacc**taatagtgatctaga**

11-43
gaattcaaccaaa**atg**gccagcaaggtgagcatcctgctgctgctgaccgtgcacctgctggccgcccagaccttcgcccaggaggtg
caccaccagaagctggtgttcttcgccgaggatgtgggtagcaacaagggtgccatcatcggtctgatggtgggtggtgtggtgatcgcc
acc**taatagtgatctaga**

3-43
gaattcaaccaaa**atg**gcccagttcctgcgcctgtgcatctggctgctggccctgggtagctgcctgctggccaccgtgcaggccgagt
tccgccacgatagcggttacgaggtgcaccaccagaagctggtgttcttcgccgaggatgtgggtagcaacaagggtgccatcatcggt
ctgatggtgggtggtgtggtgatcgccacc**taatagtgatctaga**

1-42 A42D
gaattcaaccaaa**atg**gcccagttcctgcgcctgtgcatctggctgctggccctgggtagctgcctgctggccaccgtgcaggccgatg
ccgagttccgccacgatagcggttacgaggtgcaccaccagaagctggtgttcttcgccgaggatgtgggtagcaacaagggtgccatc
atcggtctgatggtgggtggtgtggtgatc**gat****taatagtgatctaga**

1-42 A42R
gaattcaaccaaa**atg**gcccagttcctgcgcctgtgcatctggctgctggccctgggtagctgcctgctggccaccgtgcaggccgatg
ccgagttccgccacgatagcggttacgaggtgcaccaccagaagctggtgttcttcgccgaggatgtgggtagcaacaagggtgccatc
atcggtctgatggtgggtggtgtggtgatc**cgc****taatagtgatctaga**

1-42 A42W
gaattcaaccaaa**atg**gcccagttcctgcgcctgtgcatctggctgctggccctgggtagctgcctgctggccaccgtgcaggccgatg
ccgagttccgccacgatagcggttacgaggtgcaccaccagaagctggtgttcttcgccgaggatgtgggtagcaacaagggtgccatc
atcggtctgatggtgggtggtgtggtgatc**tgg****taatagtgatctaga**

11-42 E11A
gaattcaaccaaa**atg**gcccagttcctgcgcctgtgcatctggctgctggccctgggtagctgcctgctggccaccgtgcaggcc**gcc**g
tgcaccaccagaagctggtgttcttcgccgaggatgtgggtagcaacaagggtgccatcatcggtctgatggtgggtggtgtggtgatcg
cc**taatagtgatctaga**

3-42 E3A
gaattcaaccaaa**atg**gcccagttcctgcgcctgtgcatctggctgctggccctgggtagctgcctgctggccaccgtgcaggcc**gcc**t
tccgccacgatagcggttacgaggtgcaccaccagaagctggtgttcttcgccgaggatgtgggtagcaacaagggtgccatcatcggt
ctgatggtgggtggtgtggtgatcgcc**taatagtgatctaga**
