# Supplementary material for: Systematic Aβ Analysis in Drosophila Reveals High Toxicity for the 1-42, 3-42 and 11-42 Peptides, and Emphasizes N- and C-Terminal Residues
Source: PLoS One. 2015 Jul 24;10(7):e0133272. doi: 10.1371/journal.pone.0133272 (PMC4514787; doi:10.1371/journal.pone.0133272)
Supplement: S1 Table — (A) Median lifespan for different Aβ transgenes, including N-terminal aa mutants, total number of flies assayed, number of independent lifespan assays and significance versus Oregon-R (control). (B) Equivalent data for C-terminal aa mutations. (PDF) [file pone.0133272.s009.pdf]

Supplementary Table 1

A. Median lifespan of different Aβ peptides

Genotype	Median lifespan (days)	Significance vs Oregon-R (control)	Total number of flies assayed	Independent lifespan assays
1-37	42	****	116	3
1-38	37	****	113	3
1-39	28	**	106	3
1-40	35	**	175	4
1-41	30	*	158	4
1-42	9	****	168	3
1-43	25,5	****	168	3
3-42	9	****	128	3
11-42	14	****	162	4
3-43	26	****	161	4
11-43	30	ns	159	4
3-42 E3A	9	****	163	3
11-42 E11A	23	****	171	4
Oregon-R	30	-	195	4

B. Median lifespan of the C-terminal Aβ mutants

Genotype	Median lifespan (days)	Significance vs Oregon-R (control)	Significance vs each other	Total number of flies assayed	Independent lifespan assays
1-42	7	****		121	3
1-42 A42D	25	****	D vs R: *	231	5
1-42 A42R	25	****		244	5
1-42 A42W	15	****	W vs D or R:****	211	5
Oregon-R	28	-		227	5
