# Supplementary material for: Systematic Aβ Analysis in Drosophila Reveals High Toxicity for the 1-42, 3-42 and 11-42 Peptides, and Emphasizes N- and C-Terminal Residues
Source: PLoS One. 2015 Jul 24;10(7):e0133272. doi: 10.1371/journal.pone.0133272 (PMC4514787; doi:10.1371/journal.pone.0133272)
Supplement: S2 Table — Concentration of soluble and insoluble Aβ in fly head extracts, as measured by the Meso Scale Discovery (MSD) immunoassay. n/a = not assayed; d = dead at time-point. (PDF) [file pone.0133272.s010.pdf]

Supplementary Table 2. Comparison of mean Aβ protein concentration values in soluble and insoluble fraction

Genotype	Mean Soluble Aβ (ng/ml per fly)			Mean Insoluble Aβ (ng/ml per fly)			Ratio Insol/Sol (rounded to the closest integer)			Total Aβ x / total Aβ 1-40 (rounded to the closest integer)		
	d1	d10	d20	d1	d10	d20	d1	d10	d20	d1	d10	d20
1-37	3,4	0,8	1,1	5,5	7,1	2,8	2	9	3	1	1	1
1-38	1,7	1,5	0,7	2,1	2,8	2,1	1	2	3	1	1	0,4
1-39	2,4	3,0	1,6	2,9	5,3	7,9	1	2	5	1	1	1
1-40	3,3	3,0	1,9	3,9	2,8	4,7	1	1	2	1	1	1
1-41	2,0	0,8	1,0	2,3	1,6	2,5	1	2	3	1	0,4	1
1-42	1,4	2,4	d	56,5	86,3	d	39	35	d	8	15	d
1-43	3,1	1,4	2,3	5,0	12,6	17,1	2	9	7	1	2	3
3-42	0,9	4,1	d	53,8	94,7	d	62	23	d	8	17	d
11-42	1,5	0,6	d	9,0	18,9	d	6	33	d	1	3	d
3-43	2,2	0,8	1,1	3,1	9,9	8,8	1	12	8	1	2	1
11-43	n/a	n/a	n/a	n/a	n/a	n/a	n/a	n/a	n/a	n/a	n/a	n/a
3-42 E3A	0,5	1,7	d	45,8	70,0	d	93	42	d	6	12	d
11-42 E11A	0,3	0,3	0,4	4,3	6,5	7,6	17	19	22	1	1	1
1-42 A42D	3,4	4,1	3,0	11,8	12,7	10,8	3	3	4	2	3	2
1-42 A42R	3,7	4,3	3,1	11,5	16,2	14,4	3	4	5	2	4	3
1-42 A42W	3,8	2,8	1,7	11,2	8,5	8,6	3	3	5	2	2	2
Oregon-R	0,4	0,1	0,2	1,2	0,8	2,2	-	-	-	-	-	-
